# Exercise therapy improves eGFR, and reduces blood pressure and BMI in non-dialysis CKD patients: evidence from a meta-analysis

**DOI:** 10.1186/s12882-019-1586-5

**Published:** 2019-10-29

**Authors:** Lijun Zhang, Yangyang Wang, Lianlian Xiong, Yanfang Luo, Zhijun Huang, Bin Yi

**Affiliations:** 1 Department of Nephrology, The Third Xiangya Hospital, Central South University, 138 Tongzipo Road, Changsha, 410013 Hunan China; 2 Center of Clinical Pharmacology, The Third Xiangya Hospital, Central South University, Changsha, Hunan China

**Keywords:** Exercise therapy, Chronic kidney disease, Randomized controlled trial, Meta-analysis

## Abstract

**Background:**

Patients with chronic kidney disease (CKD) have a high prevalence of cardiovascular diseases, which often lead to physical inactivity that correlates with CKD exacerbation. The benefits of regular exercise to cardiovascular health have been well established in healthy population and highly suggestive in patients with CKD. To further strengthen the evidence base for the management of CKD, this meta-analysis was performed to systematically evaluate the effects of exercise therapy on renal function, blood pressure, blood lipid and body mass index (BMI) in non-dialysis CKD patients.

**Methods:**

This meta-analysis was conducted following a previous protocol. Randomized controlled trials (RCTs) examining the effects of exercise therapy in non-dialysis CKD patients were searched in Pubmed, Embase, Cochrane Library, and three major Chinese biomedical databases (CNKI, WANGFANG and VIP) from their start date to October 30th, 2018. The Cochrane systematic review methods were applied for quality assessment and data extraction, and Revman version 5.3 was used for systematic review and meta-analysis.

**Results:**

13 RCTs, representing 421 patients with non-dialysis CKD, were included in this meta-analysis. Compared to the controls, exercise therapy brought an increase in eGFR (MD = 2.62, 95% CI:0.42 to 4.82, *P* = 0.02, I^2^ = 22%), and decreases in systolic blood pressure (SBP) (MD = -5.61, 95% CI:-8.99 to − 2.23, *P* = 0.001, I^2^ = 44%), diastolic blood pressure (DBP) (MD = -2.87, 95% CI:-3.65 to − 2.08, *P* < 0.00001, I^2^ = 16%) and BMI (MD = -1.32, 95% CI:-2.39 to − 0.25, *P* = 0.02, I^2^ = 0%) in non-dialysis CKD patients. Exercise therapy of short-term (< 3 months) decreased triglyceride (TG) level (*P* = 0.0006). However, exercise therapy did not significantly affect serum creatinine (SCr), total cholesterol (TC), high density lipoprotein (HDL) or low density lipoprotein (LDL) in non-dialysis CKD patients.

**Conclusion:**

Exercise therapy could benefit non-dialysis CKD patients by increasing eGFR while reducing SBP, DBP and BMI. Additionally, short-term intervention of exercise could decrease TG.

## Background

Chronic kidney disease (CKD) has become a worldwide public health issue threatening human health. With the increasing incidence of hypertension, diabetes and obesity, the overall prevalence of CKD has also risen significantly. More than 119 million Chinese adults had been estimated to have CKD by 2012 [1]. Because of gradual muscle strength loss, uremic milieu and dialysis complications, the activity levels of CKD patients are markedly lower than those of healthy individuals, further deteriorating their basic activities of daily living and life quality, and increasing all-cause mortality [2, 3].

Traditional risk factors, such as hypertension, diabetes, dislipidemia and obesity, promote CKD progression. Studies have shown that moderate-intensity exercises provide cardiovascular protection and metabolic benefits not only for healthy individuals, but also for patients with hypertension and coronary heart disease (CHD) [4–6]. The impact of exercise on the prognostic factors of CKD have also been widely investigated. Aoike et al. evaluated the effects of aerobic training on overweight non-dialysis-dependent CKD patients, and found that exercise improved blood pressure (BP), but not renal function or body mass index (BMI) in these patients [7]. Nonetheless, other studies have reported lack of impact of exercise on BP in CKD patients [8, 9]. Toyama et al. showed that exercise therapy could modify lipid metabolism and improve estimated glomerular filtration rate (eGFR) in patients with cardiovascular disease and CKD [9]. Greenwood et al. confirmed that moderate-intensity exercise provided benefits in kidney function and BMI for patients with stage 3–4 CKD [10]. There are still discrepancies among these studies, and these studies mainly focused on end-stage renal disease (ESRD) patients receiving regular dialysis. In order to strengthen the theoretical basis for the management of CKD, we performed here a meta-analysis to systematically evaluate the effects of exercise therapy on renal function, blood pressure, blood lipids and BMI in non-dialysis CKD (stage 2–5) patients.

## Materials and methods

### Literature search strategy

We performed a systematic literature search (Pubmed, Embase, Cochrane Library, CNKI, WANGFANG, VIP) to identify literatures published from the establishment of these databases to October 30th, 2018 in either English or Chinese. MeSH-terms or keywords used in the literature search included “exercise therapy”, “aerobic exercise”, “exercise”, “rehabilitation”, “exercise training”, “renal insufficiency”, “chronic kidney disease”, “chronic renal failure”, “pre-dialysis”, “non-dialysis”, “eGFR”, “glomerular filtration rate”, “creatinine”, “randomized controlled trial”, “blood pressure”, “total cholesterol”, “triglycerides”, “high density protein”, “low density lipoprotein” or “serum lipids”. Additional studies were screened through a manual search of reference lists of these literatures. The literature search was conducted by two of the authors (L.Z. and Y.W.) independently.

### Inclusion criteria

This systematic search and meta-analysis was performed and reported according to the Preferred Reporting Items for Systematic Reviews and Meta-Analyses (PRISMA) guidelines [11] and checklist (Additional file 1: Checklist). Only randomized controlled trials (RCTs) were included. The following inclusion criteria were fulfilled: 1) adult human (> 18 years); 2) non-dialysis CKD patients (stage 2–5); 3) aerobic exercise and/or resistance exercise; 4) exercise at least once a week for more than one month; 5) studies describing the effects of exercise on eGFR, BP or blood lipids; 6) test method was described accurately and the criteria used to judge the expected outcomes were listed clearly.

### Exclusion criteria

Exclusion criteria were: 1) non-randomized controlled trials, such as observational studies, reviews, meta-analysis, short reports, research programs, and animal trials; 2) RCTs whose study population was dialysis CKD patients; 3) RCTs with inappropriate statistical methods; 4) RCTs with incomplete data; 5) duplicated articles with poor quality; 6) RCTs whose data were not described by mean and standard deviation.

### Data extraction

Quantitative data were extracted from the included articles independently by two authors (L.Z. and Y.W.), and differences in opinion were resolved by joint discussion. The extracted contents included author, year of publication, source of literature, research design, duration, sample size, general situation of subjects, intervention measures and other general information. The main extracted contents included eGFR, creatinine, BP, blood lipid, and BMI.

### Quality assessment

Following our previously published protocol [12], two researchers (L.Z. and Y.W.) performed quality assessment of the selected RCTs independently. The senior author (Y.B.) was consulted for any discrepancy between the two researchers to reach consensus. According to the Cochrane Collaboration for Systematic Reviews of Interventions, the following guidelines were applied to assess all included RCTs: properly used randomization; properly used allocation concealment; properly used methods of blinding; exclusion of data introducing incomplete-data bias; exclusion of data introducing selection bias; and exclusion of data introducing other potential biases.

### Statistical analysis

Statistical analyses were carried out according to a previous published plan [12]. Results from 13 included RCTs were pooled and analyzed via Revman Review Manager version 5.3. Mean deviation (MD) and standard deviation (SD) with 95% confidence interval (CI) were used to express continuous variables. The χ2 test was used for heterogeneity assessment. The fixed effect model, the sensitive analysis, or the random effect model of meta-analysis was used in the absence of clinical heterogeneity (*P* ≥ 0.05 and I^2^ ≤ 50%), in the presence of substantial heterogeneity among studies (*P* < 0.05 and I^2^ > 50%) or when the source of heterogeneity remained unclear, respectively. *P* < 0.05 was considered statistically significant. Publication bias, evaluated via the funnel chart, was considered minimal if the distribution of scattered points was symmetrical. Besides the evaluation of overall risk, analyses were also undertaken in subgroups to compare the impact of intervention duration on outcomes.

## Results

### Summary of literature search and study selection

**Fig. 1:**
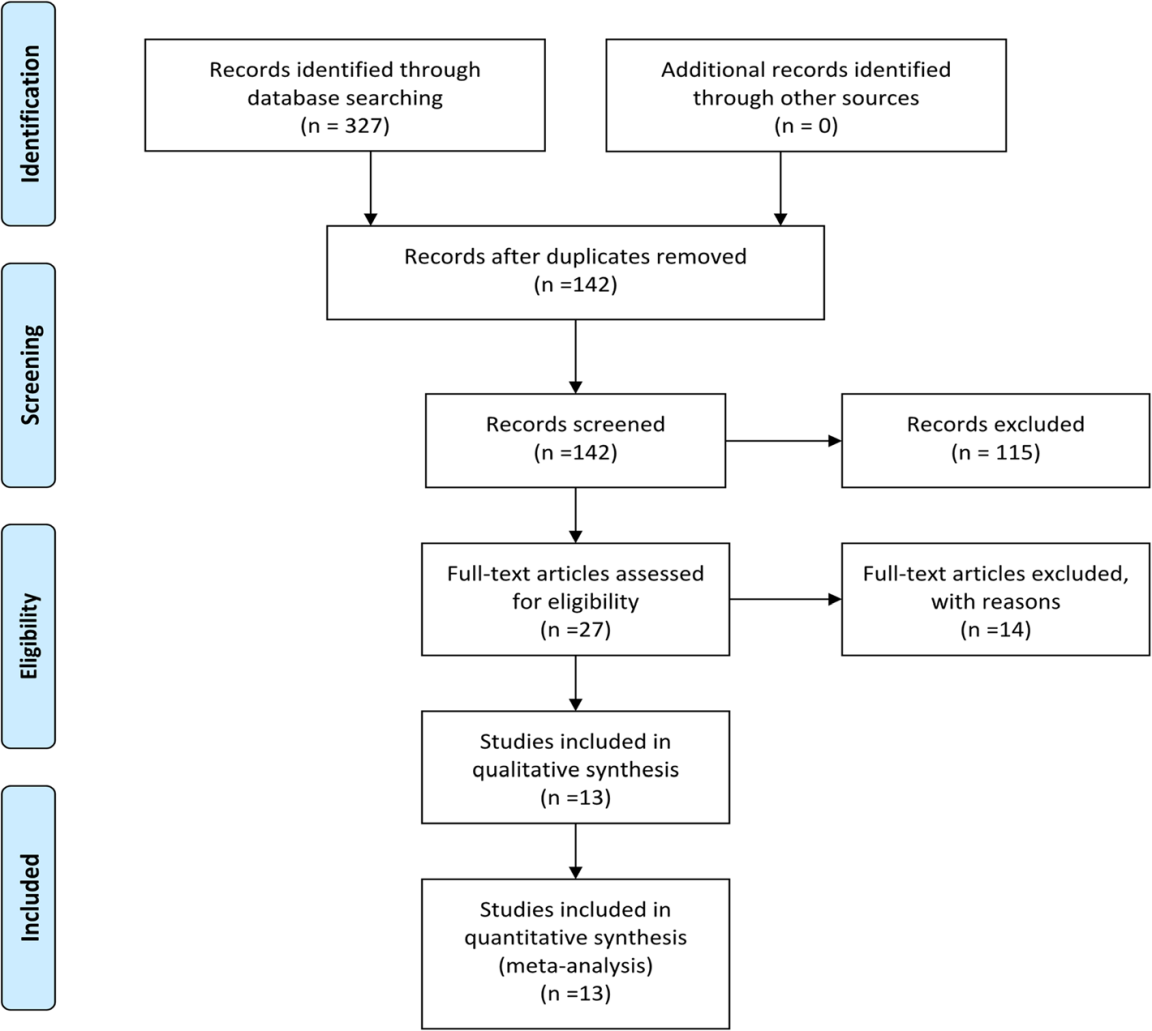
PRISMA flow diagram for systematic review and meta-analysis

**Fig. 2:**
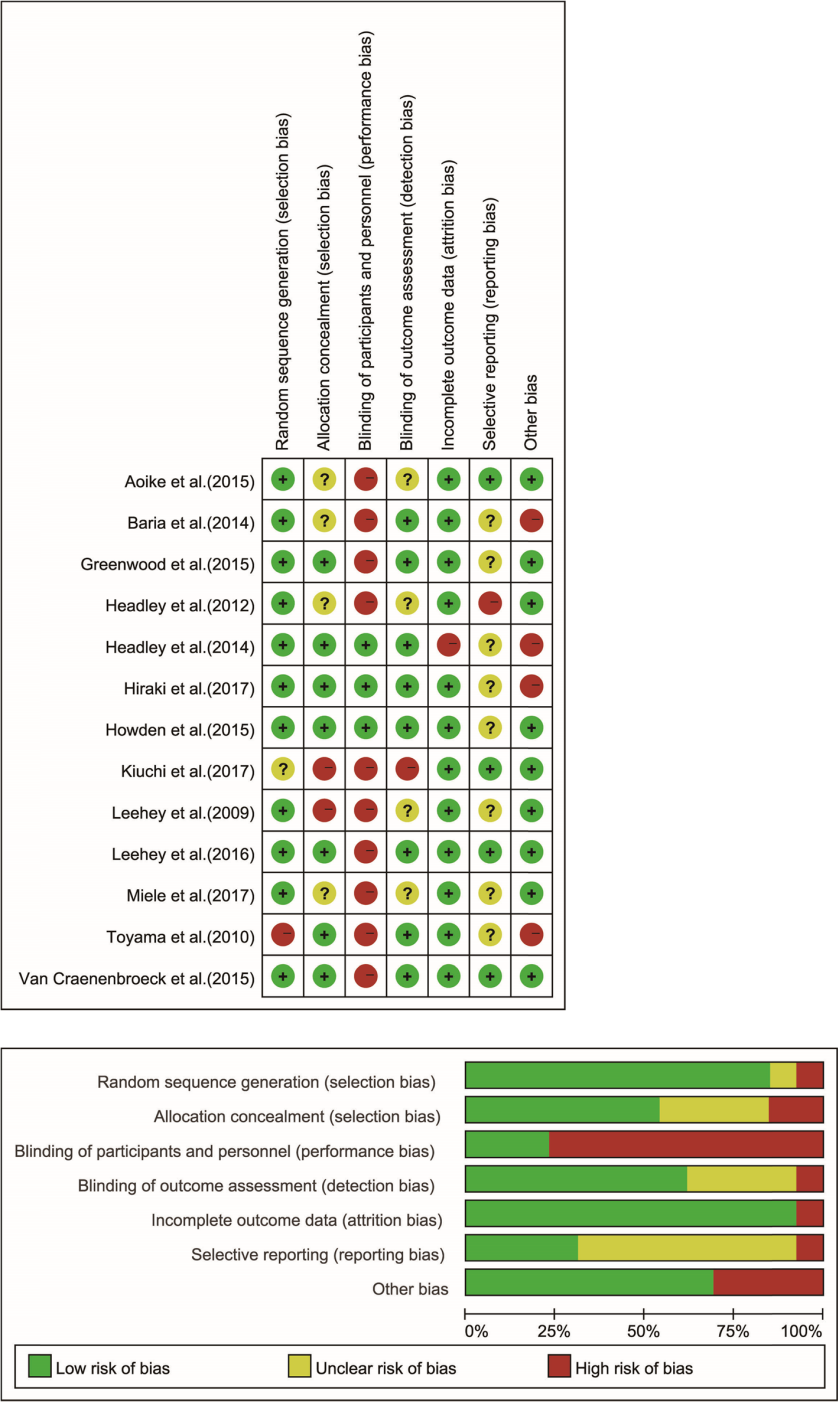
Methodological quality of the included studies

We identified 327 articles via literature search. 27 studies remained after reviewing title and abstract, and excluding non-RCTs, duplicate publications or nonclinical research. We further examined the full text of these 27 studies and excluded those involved exercise therapy of longer than 12 months or heterogeneous patient populations, or failed to provide qualified endpoints or complete data for our meta-analysis. Ultimately 13 RCTs [7–10, 13–21] were included in our meta-analysis (all published in English), involving a total of 421 participants (Experimental/Control: 212/209)(Fig.1). There were two follow-up outcomes in several RCTs [8, 10, 16, 20, 21]. The characteristics of all included RCTs were summarized in Table 1 and the methodological quality of the included RCTs were shown in Fig. 2.

**Table 1:** Baseline characteristics of the included studies

Author and Year	Study population	Participants (T/C)	Intervention	Study duration	Parameters	Main results
Miele 2017	CKD 3	46(25/21)	Aerobic exercise three times a week	16 Weeks	eGFR; TC; TG LDL; HDL; BMI	No significant difference in eGFR between the two groups
Hiraki 2017	CKD3–4	28(14/14)	Aerobic and resistance exercise	12 Months	eGFR	No significant difference in eGFR between the two groups
Kiuchi 2017	CKD3–5	40(20/20)	Moderate exercise, 150 min a week, gradually increasing to 300 min a week	12 Months	eGFR; SCr; Blood pressure	Decreased SCr; Improved eGFR
Leehey 2016	CKD 2–4	32(14/18)	Aerobic and resistance exercises three days a week lasted for 12 weeks, followed by Home-based exercises for 40 weeks.	12 Weeks 52 Weeks	eGFR; Systolic blood pressure; TC; TG LDL; HDL; BMI	NO change eGFR, systolic blood pressure and blood lipid both after 12 and 52 Weeks
Aoike 2015	CKD3–4	29(14/15)	Aerobic exercise, 3 times a week	12 weeks	GFR; SCr; Blood pressure; BMI	Decreased blood pressure and improved renal function
Greenwood 2015	CKD3–4	18(8/10)	Aerobic and resistance exercises, 3 days a week	6 Months 12 Months	eGFR; SCr; Blood pressure; TC; LDL; HDL; BMI	eGFR increased slightly after 6 months, Improvement mean rate of change in eGFR and SCr after 12 months, but no change blood pressure, blood lipid and creatinine
Howden 2015	CKD3–4	72(36/36)	Aerobic and resistance exercises, 150 min a week	6 Months 12 Months	eGFR; SCr; Blood pressure; TC; LDL; HDL; BMI	NO change in eGFR and blood pressure after both 6 and 12 Months
Van Craenenbroeck 2015	CKD3–4	40(19/21)	Home-based aerobic exercises,4 times a day, 10 min each time	3 Months	eGFR; Blood pressure; TC; LDL; HDL; BMI	NO improvement in eGFR and blood pressure
Baria 2014	CKD3–4	19(10/9)	Aerobic exercise, 3 times a week	12 Weeks	eGFR; Blood pressure; BMI	Decreased Blood pressure and improved eGFR
Headley 2014	CKD 3	46(25/21)	Aerobic exercise, 3 times a week	16 Weeks	Blood pressure; BMI	NO change in blood pressure
Headley 2012	CKD2–4	21(10/11)	Aerobic exercise, 3 times a week for 24 weeks, followed by resistance exercise, 2 times a week for 24 weeks	24 Weeks 48 Weeks	eGFR; Blood pressure; TC; TG LDL; HDL; BMI	TG increased at 24 weeks, but no change at 48 weeks. TC and LDL increased at 24 and 48 weeks, but no change eGFR, blood pressure and HDL
Toyama 2010	CKD2–4	19(10/9)	Aerobic exercise once a week	12 Weeks	eGFR	Increased eGFR
Leehey 2009	CKD2–4	11(7/4)	Aerobic exercise, 3 times a week for 6 weeks, followed by home-based exercise for 18 weeks	6 Weeks 24 Weeks	eGFR; SCr; Blood pressure; TC; TG; LDL; HDL	Systolic blood pressure decreased slightly at 6 weeks, but GFR, blood lipid and blood pressure did not improve; no improvement between the two groups at 24 weeks

Note: *T* Treatment group, *C* Control group, *eGFR* estimated glomerular filtration rate (According to MDRD formula). *TC* Total cholesterol, *TG* Triglyceride, *LDL* Low density lipoprotein, *HDL* High density lipoprotein, *BMI* Body mass index

### The impact of exercise therapy on indexes of renal function

#### eGFR

**Fig. 3:**
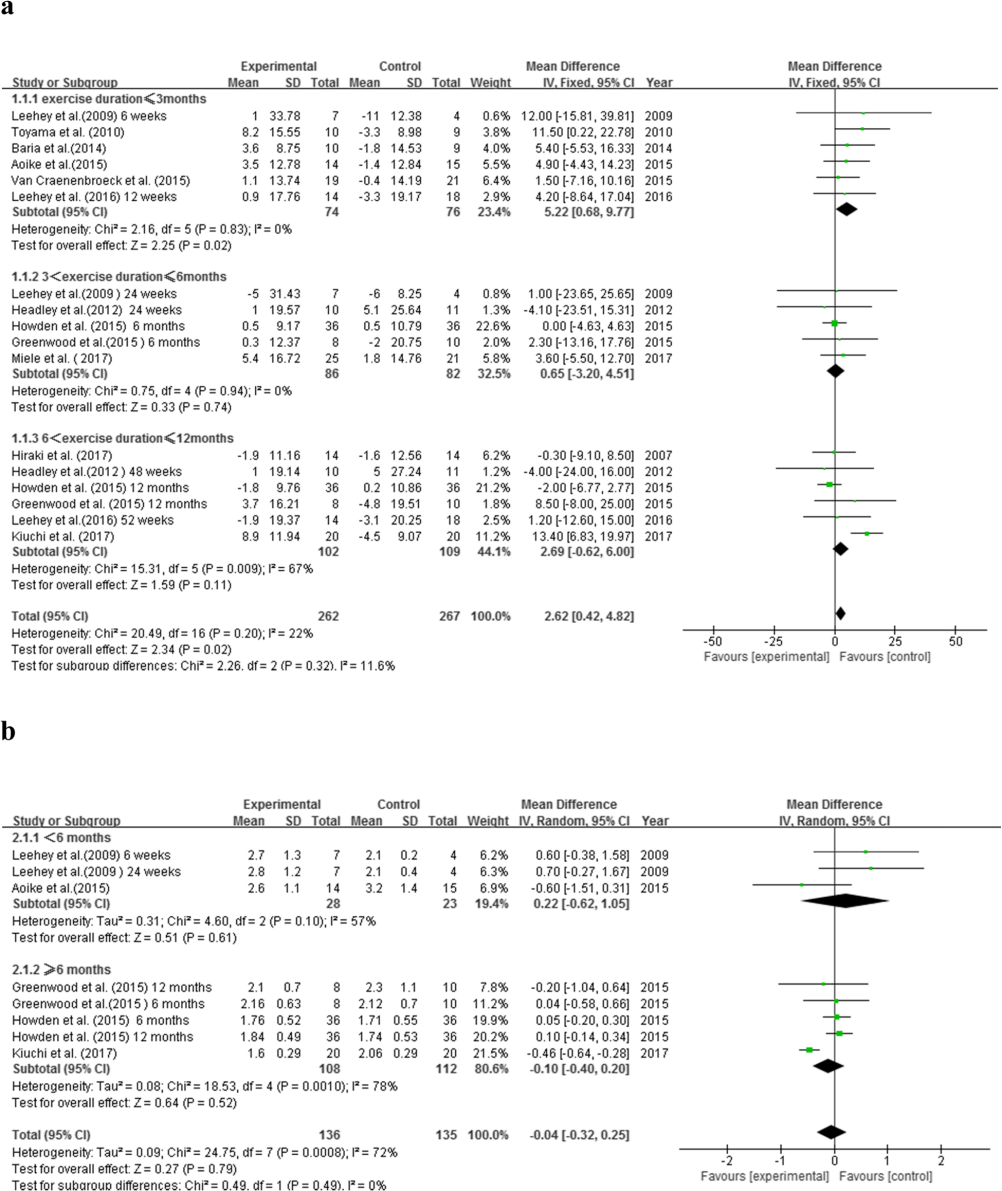
Effects of exercise therapy on indexes of renal function. **a**. Effect on eGFR based on duration of exercise. **b**. Effect on SCr based on duration of exercise

12 RCTs involving 529 participants (E/C: 262/267) reported eGFR as primary or secondary outcome (Fig. 3a). eGFR was calculated using the CKD-EPI creatinine equation [7, 10, 15, 18] or the MDRD formula [8, 9, 13, 14, 16, 17, 20, 21]. Duration of exercise intervention varied between 6 weeks [21] and 12 months [8, 10, 14–16]. There was no significant difference in heterogeneity among the 12 RCTs (*P* = 0.20, I^2^ = 22%). Our results showed that exercise intervention could bring an average increase of 2.62 ml/min/1.73m^2^ in eGFR in non-dialysis CKD patients, and the difference was statistically significant (MD = 2.62, 95% CI:0.42 to 4.82, *P* = 0.02). When these participants were further stratified based on the duration of exercise intervention, we found that compared to non-exercise subjects, eGFR was significantly increased (by 5.22 ml/min/1.73m^2^) with short-term exercise (< 3 months) (MD = 5.22, 95% CI: 0.68 to 9.77, *P* = 0.02), but not with 3–6 months of exercise (MD = 0.65, 95% CI: − 3.20 to 4.51, *P* = 0.74) or 6–12 months of exercise (MD = 2.69, 95% CI: − 0.62 to 6.00, *P* = 0.11).

#### Scr

Only 5 RCTs reported sufficient data to evaluate the effect of exercise therapy on Scr, involving 271 participants (E/C: 136/135). Heterogeneity assessment indicated high study heterogeneity (*P* = 0.0008, I^2^ = 72%). Using the random effect model, we found that there was no significant difference in Scr between the intervention and the control groups (MD = -0.04, 95% CI: − 0.32 to 0.25, *P* = 0.79). Subgroup analysis based on exercise duration showed no significant improvement of Scr in participants who received exercise intervention of less than 6 months (MD = 0.22, 95% CI: − 0.62 to 1.05, *P* = 0.61) or exercise intervention of 6–12 months (MD = -0.10, 95% CI: − 0.40 to 0.20, *P* = 0.52) (Fig. 3b).

### The impact of exercise therapy on indexes of blood pressure

#### Systolic blood pressure

**Fig. 4:**
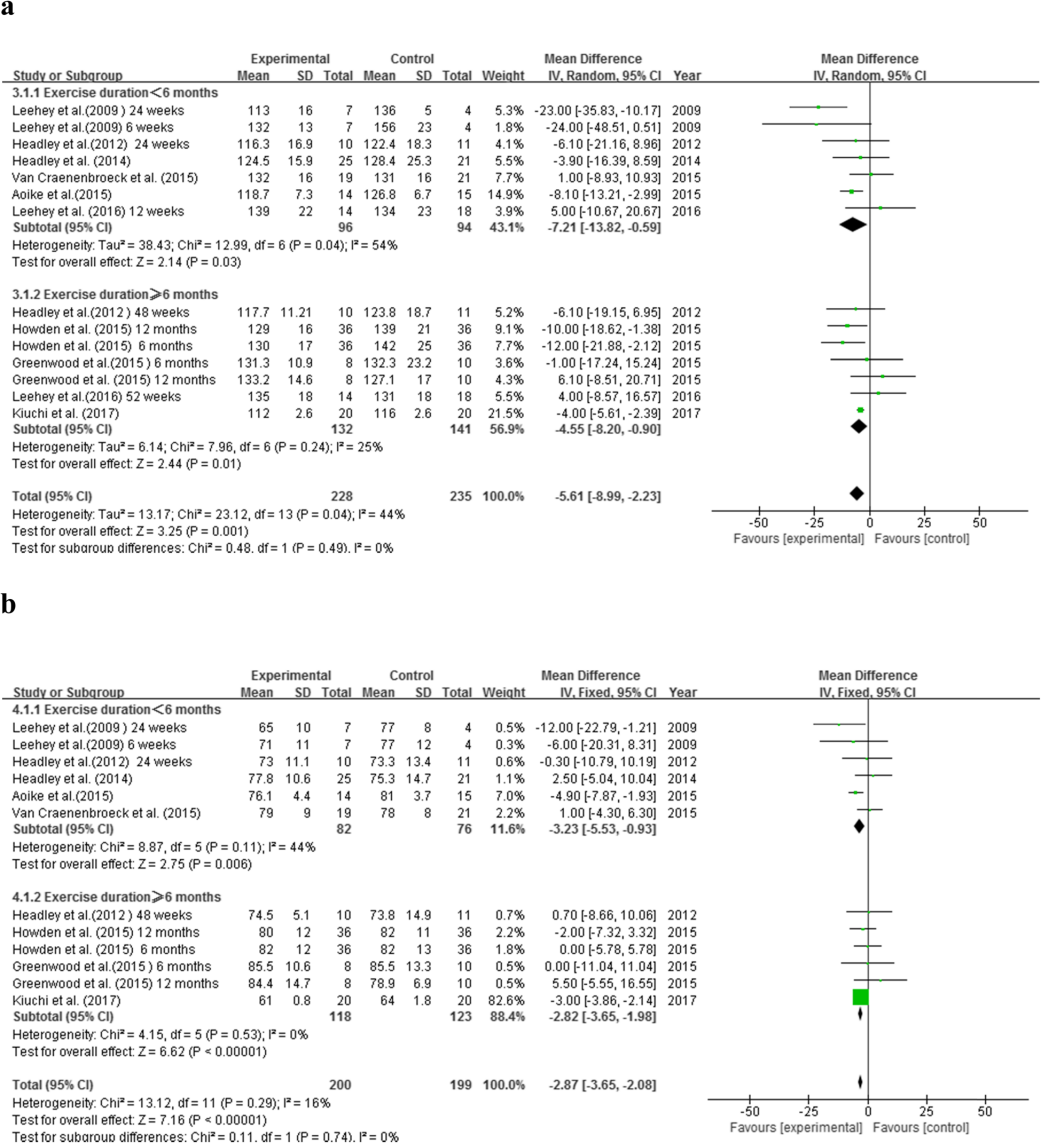
Effects of exercise therapy on indexes of blood pressure. **a.** Effect on systolic blood pressure based on duration of exercise. **b.** Effect on diastolic blood pressure based on duration of exercise

9 RCTs examined systolic blood pressure (SBP) and 14 groups of data (involving 463 participants, E/C: 228/235) were analyzed. A mild statistical heterogeneity was noted among the included studies (*P* = 0.04, I^2^ = 44%). Meta-analysis using the random effect model showed that exercise therapy could markedly reduce SBP by 5.61 mmHg (MD = -5.61, 95% CI: − 8.99 to − 2.23, *P* = 0.001) in non-dialysis CKD patients. Subgroup analysis showed that SBP was significantly decreased regardless of the duration of exercise intervention. Compared to non-exercise subjects, SBP was decreased by 7.21 mmHg (MD = -7.21, 95% CI: − 13.82 to − 0.59, *P* = 0.03) in participants who received exercise therapy of less than 6 months, and by 4.55 mmHg (MD = -4.55, 95% CI: − 8.20 to − 0.90, *P* = 0.01) in those who received exercise therapy of 6–12 months (Fig.4a).

Diastolic blood pressure

8 RCTs including 12 groups of data were analyzed (*N* = 399, E/C: 200/199). Heterogeneity was not obvious (*P* = 0.29, I^2^ = 16%), so the fixed-effect approach was applied. Exercise therapy decreased diastolic blood pressure (DBP) of non-dialysis CKD patients by 2.87 mmHg (MD = -2.87, 95% CI: − 3.65 to − 2.08, *P* < 0.00001). Additionally, this DBP improvement was independent of the duration of exercise intervention. DBP was decreased by 3.23 mmHg in subjects receiving less than 6 months of exercise therapy (MD = -3.23, 95% CI: − 5.53 to − 0.93, *P* = 0.006) and by 2.82 mmHg (MD = -2.82, 95% CI: − 3.65 to − 1.98, P < 0.00001) in subjects receiving 6–12 months of exercise therapy (Fig. 4b).

### The impact of exercise therapy on indexes of **blood lipids**

#### TC

**Fig. 5:**
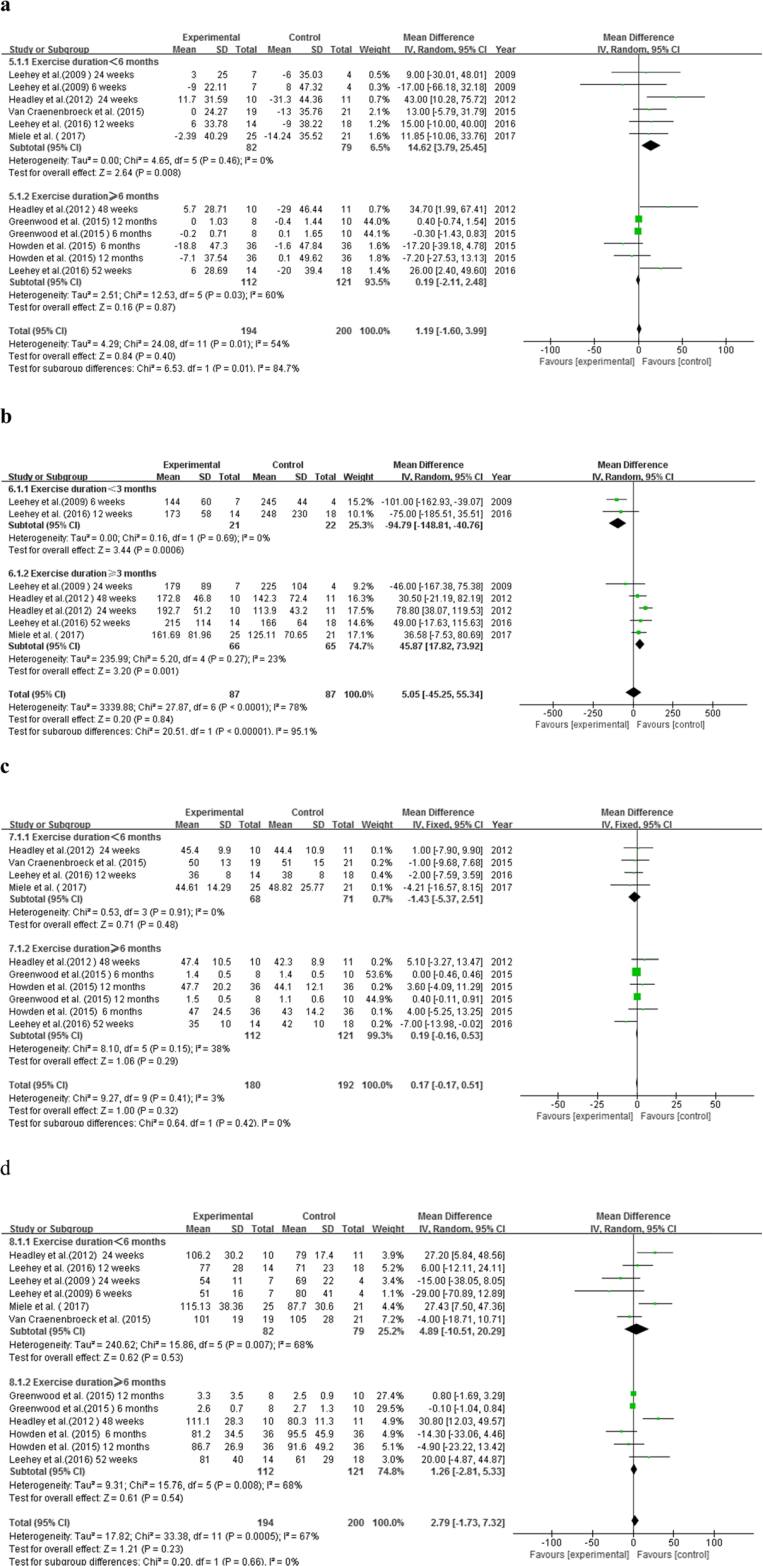
Effects of exercise therapy on indexes of blood lipids. **a.** Effect on TC based on duration of exercise. **b.** Effect on TG based on duration of exercise. **c**. Effect on HDL based on duration of exercise. **d.** Effect on LDL based on duration of exercise

The effect of exercise therapy on TC was assessed in 7 RCTs involving 394 participants (E/C: 194/200). There was a high level of heterogeneity among these studies (*P* = 0.01, I^2^ *=* 54%). Meta-analysis showed no significant difference in TC level between the experimental and the control groups (MD = 1.19, 95% CI: − 1.60 to 3.99, *P* = 0.40). Nonetheless, subgroup analysis indicated a significantly increased TC level in non-dialysis CKD patients who received exercise therapy of less than 6 months (MD = 14.62, 95% CI: 3.79 to 25.45, *P* = 0.008), but not in those who received exercise therapy of 6–12 months (MD = 0.19, 95% CI: − 2.11 to 2.48, *P* = 0.87) (Fig. 5a).

#### TG

4 RCTs, including 7 groups of data, described the effect of exercise therapy on TG and were analyzed (*N* = 174, E/C: 87/87). The random-effect model was used because obvious heterogeneity (*P* < 0.0001, I^2^ = 78%). No significant difference in TG level was found between the experimental and the control groups (MD = 5.05, 95% CI: − 45.25 to 55.34, *P* = 0.84). However, exercise intervention of less than 3 months markedly decreased TG (MD = -94.79, 95% CI: − 148.81 to − 40.76, *P* = 0.0006), while exercise intervention of 3–12 months increased TG (MD = 45.87, 95% CI: 17.82 to 73.92, *P* = 0.001) in non-dialysis CKD patients (Fig. 5b).

#### HDL

12 groups of data from 7 RCTs were included and analyzed (*N* = 372, E/C: 180/192). Heterogeneity was statistically low (*P* = 0.41, I^2^ = 3%). Exercise intervention did not significantly affect HDL level in non-dialysis CKD patients (MD = 0.17, 95% CI: − 0.17 to 0.51, *P* = 0.32), even after participants were stratified based on the duration of the exercise intervention (MD = -1.43, 95% CI: − 5.37 to 2.51, *P* = 0.48 for subject receiving less than 6 months of exercise therapy, and MD = 0.19, 95% CI: − 0.16 to 0.53, *P* = 0.29 for those receiving 6–12 months of exercise therapy, respectively) (Fig. 5c).

#### LDL

7 RCTs, including 12 groups of data (*N* = 394, E/C: 194/200), examined LDL levels and were analyzed. These studies presented high heterogeneity (*P* = 0.0005, I^2^ = 67%) and no significant difference was found in LDL level between the experimental and the control groups (MD = 2.79, 95% CI: − 1.73 to 7.32, *P* = 0.23), even after stratification based on the duration of exercise intervention (Fig. 5d).

### The impact of exercise therapy on **BMI**

**Fig. 6:**
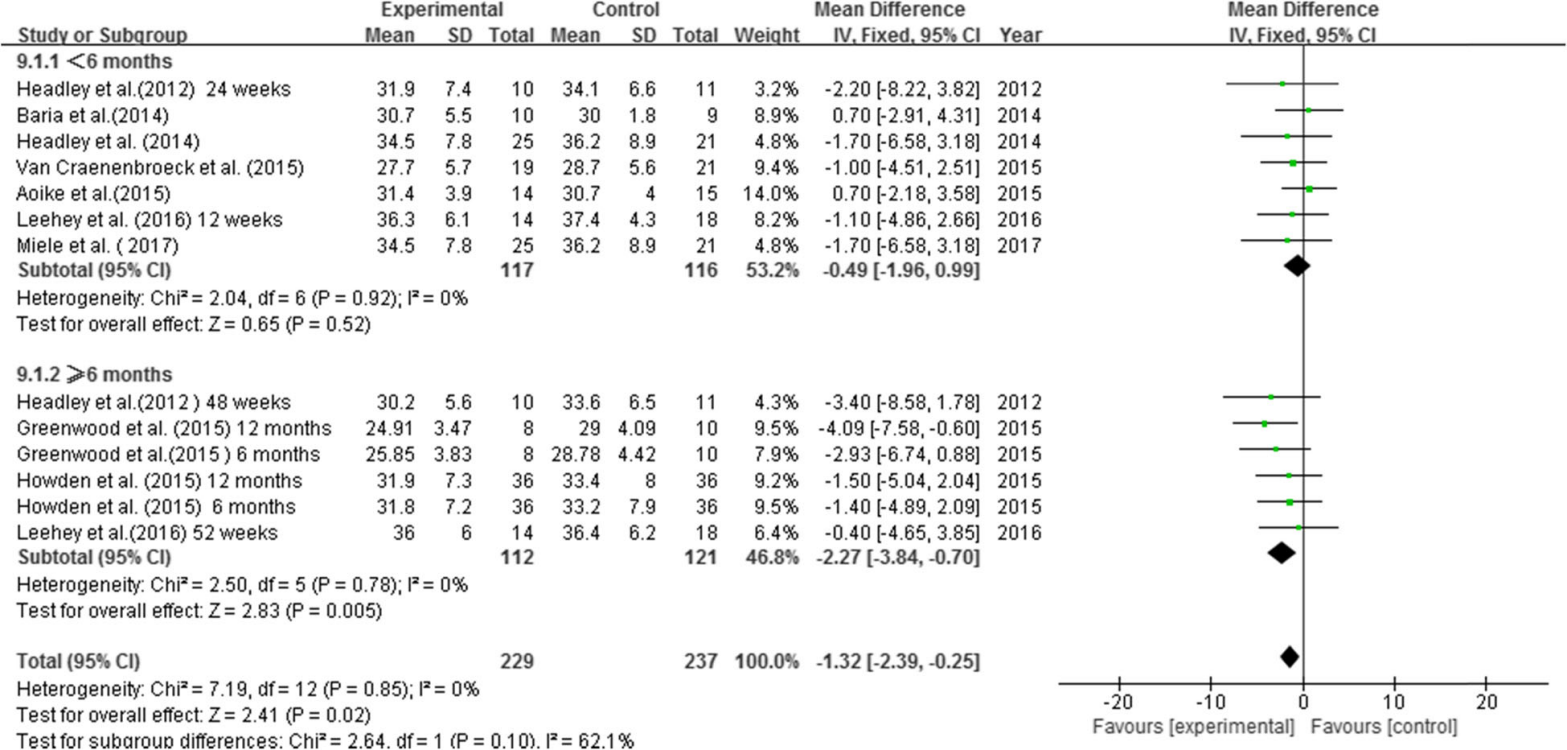
Effects of exercise therapy on BMI

A total of 13 groups of data from 9 RCTs were included for comparison (*N* = 466, E/C: 229/237) and there was low heterogeneity among the studies (*P* = 0.85, I^2^ = 0%). Meta-analysis using the fixed-effect model showed that exercise therapy significantly reduced BMI in non-dialysis CKD patients (MD = -1.32, 95% CI: − 2.39 to − 0.25, *P* = 0.02). Subgroup analysis revealed that compared to the controls, BMI of non-dialysis CKD patients was significantly reduced (by 2.27 kg/m^2^) in those who received 6–12 months of exercise intervention (MD = -2.27, 95% CI: − 3.84 to − 0.70, *P* = 0.005), but not in those who received less than 6 months of exercise intervention (MD = -0.49, 95% CI: − 1.96 to 0.99, *P* = 0.52) (Fig. 6).

### Sensitivity analysis and publication bias

We performed sensitivity analysis on all 13 included RCTs using Leave-One-Out methods. Variations of the combined effects after excluding each indicator variable was relatively small, indicating the robustness of our meta-analytic results. Funnel plots showed that studies assessing eGFR, creatinine, blood pressure, blood lipid, BMI were symmetrically distributed on the funnel plots, suggesting small publication biases (Additional files 2, 3, 4, 5, 6, 7, 8, 9 and 10).

### Quality assessment

Of the included 13 RCTs, potential bias was mainly originated from blinding of participants and personnel. Because blinding of participants and personnel is very challenging in RCTs involving exercise intervention, high risk of performance bias was granted to more than 75% of the studies [7, 9, 10, 13, 15–18, 20, 21]. Risk for detection bias was unclear in 4 RCTs [7, 13, 20, 21], and was scored as high in Kiuchi study only [15]. Baseline data such as age, sex, blood pressure, blood lipid and BMI were examined by equilibrium test, and the differences were not statistically significant (*P* > 0.05). The results of quality assessment of all literatures were summarized in Fig. 2.

## Discussion

Our meta-analysis confirms that exercise therapy reduces SBP by 5.61 mmHg and DBP by 2.87 mmHg in stage 2–5 non-dialysis CKD patients. This anti-hypertensive effect is specifically significant in participants who received exercise therapy of less than 6 months. We found that longer term exercise therapy of 6–12 months was not associated with greater anti-hypertensive effect. It is possible that with the deteriorated renal insufficiency and renal dysfunction offset some beneficial adaptations. Consistent with our results, a recent meta-analysis of Thompson [22], which focused on the effects of exercise on BP in non-dialysis CKD patients, found that exercise overall did not significantly impact SBP or 4-h ambulatory BP. Additionally, SBP benefited from exercise of 12–16 weeks or of 24–26 weeks, but not from exercise of 48–52 weeks. Susanne Heiwe [23] showed that regular exercise had significant beneficial effects on walking ability, BP and heart rate of adult CKD patients. On the other hand, Vanden Wyngaert K’s meta-analysis showed that exercise had no effect on BP in CKD patients [24], possibly because their analysis was limited to stage 3–4 CKD patients. CKD patients often have high TG and low HDL levels, which could facilitate the progression of CKD [25, 26]. We found that exercise therapy of less than 3 months significantly reduced TG, but had no effect on TC, HDL or LDL. Xiong et al. also found that physical exercise improved cardiopulmonary endurance in renal transplant recipients, but provided no benefit for blood lipids, BP or hemoglobin [27]. This might be due to the primary diseases, dietary structure, compliance with interventions and the use of lipid-lowering drugs in these patients. Further follow-up studies with larger samples and longer duration of intervention are needed to determine whether exercise therapy could improve lipid status in non-dialysis CKD patients.

Furthermore, our meta-analysis showed that exercise therapy could increase eGFR by 2.62 ml/min/1.73m^2^ in non-dialysis CKD patients. Pechter et al. [28] observed the effect of 10 years of low intensity aerobic water exercise on CKD patients, and found that 100% of exercise group survived, while 55% of patients in the control group died or needed renal replacement therapy (this study was not included because of its intervention duration was significantly different from other RCTs included in our meta-analysis). Heather et al. also showed that decreased physical activity in non-dialysis CKD patients (including kidney transplant recipients) was associated with increased mortality and adverse clinical events, including impaired renal function, increased risk in kidney replacement therapy and reduced survival rate of renal graft [29]. We speculate that exercise therapy may improve endothelium-mediated vasodilatation, attenuate the increased sympathetic nervous system activity, reduce urinary protein, improve renal blood flow and residual renal function in non-dialysis CKD patients by decreasing BP, blood lipids and BMI, and thereby delaying renal function deterioration [18, 30, 31].

There are several limitations to the conclusions of our meta-analysis that are inherent to the studies included. First, although only RCTs were involved, which reduced bias to a certain extent, the designs of the included trials were not entirely uniform. Some experimental designs and methods, such as randomization, were not clearly described. These parameters are very important in evaluating the quality of the experiment. As a result, the quality of these RCTs may be decreased. In addition, the sample size was relatively small, and the mode, intensity and frequency of exercise therapy were not completely consistent. The literatures included in this meta-analysis could not be accurately classified according to the types of exercise therapy and such subgroup analysis was not performed. Despite the limitations mentioned above, our literature collection was comprehensive, and subgroup analysis was carried out based on the duration of exercise intervention to ensure the reliability of the research results. Therefore, this study could at least provide some objective basis for the discussion of the relationship between exercise therapy and non-dialysis CKD.

## Conclusion

In conclusion, exercise therapy may be a potential strategy to improve eGFR, reduce SBP, DBP and BMI in non-dialysis CKD patients. Limited evidence from short-term studies suggests that exercise may reduce TG, but not Scr, TC, HDL or LDL. Clinical studies of kidney diseases have always focused on ESRD patients requiring renal replacement therapy, but we should pay more attention to the less studied non-dialysis CKD patients and find interventions to improve their life quality. In the future, RCTs with larger sample size, multicenter and long-term follow-up are necessary in order to clarify the impact of exercise on preventing cardiovascular complications and CKD progression in non-dialysis CKD patients. Exercise therapy could be a low-cost and convenient treatment strategy for non-dialysis CKD patients, with significant social benefits.

## Supplementary information

**Additional file 1.** Checklist.

**Additional file 2: Fig. S1.** Funnel plot of between-groups analysis for eGFR.

**Additional file 3: Fig. S2.** Funnel plot of between-groups analysis for SCr.

**Additional file 4: Fig. S3.** Funnel plot of between-groups analysis for SBP.

**Additional file 5: Fig. S4.** Funnel plot of between-groups analysis for DBP.

**Additional file 6: Fig. S5.** Funnel plot of between-groups analysis for TC.

**Additional file 7: Fig. S6.** Funnel plot of between-groups analysis for TG.

**Additional file 8: Fig. S7.** Funnel plot of between-groups analysis for HDL.

**Additional file 9: Fig. S8.** Funnel plot of between-groups analysis for LDL.

**Additional file 10: Fig. S9.** Funnel plot of between-groups analysis for BMI.

## Data Availability

The datasets generated and analyzed during the current study are available from the corresponding author on reasonable request.

## Abbreviations

BMI: Body mass index
CHD: Coronary heart disease
CKD: Chronic kidney disease
DBP: Diastolic blood pressure
ESRD: End-stage renal disease
HDL: High density lipoprotein
LDL: Low density lipoprotein
RCTs: Randomized controlled trials
SBP: Systolic blood pressure
SCr: Serum creatinine
TC: Total cholesterol
TG: Triglyceride
