# Supplementary figures and images for: Exercise therapy improves eGFR, and reduces blood pressure and BMI in non-dialysis CKD patients: evidence from a meta-analysis
Source: BMC Nephrol. 2019 Oct 29;20:398. doi: 10.1186/s12882-019-1586-5 (PMC6821004; doi:10.1186/s12882-019-1586-5)

**Additional file 2:**

**Figure S1: Funnel plot of between-groups analysis for eGFR.**

**
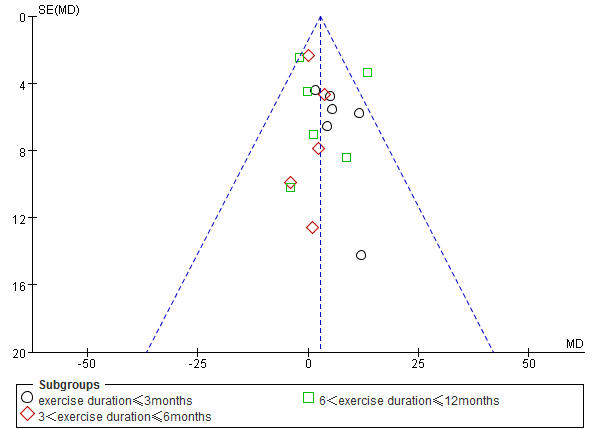
**

Supplement: Supplementary file 2 — Additional file 2: Fig. S1. Funnel plot of between-groups analysis for eGFR. [file 12882_2019_1586_MOESM2_ESM.docx]

**Additional file 3:**

**Figure S2: Funnel plot of between-groups analysis for** **SCr.**

**
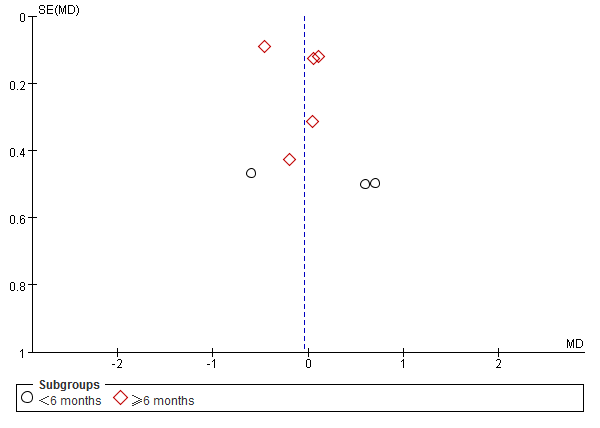
**

Supplement: Supplementary file 3 — Additional file 3: Fig. S2. Funnel plot of between-groups analysis for SCr. [file 12882_2019_1586_MOESM3_ESM.docx]

**Additional file 4:**

**Figure S3: Funnel plot of between-groups analysis for SBP.**

**
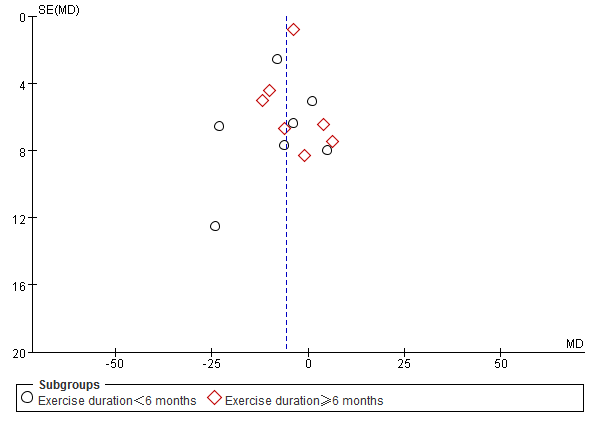
**

Supplement: Supplementary file 4 — Additional file 4: Fig. S3. Funnel plot of between-groups analysis for SBP. [file 12882_2019_1586_MOESM4_ESM.docx]

**Additional file 5:**

**Figure S4: Funnel plot of between-groups analysis for DBP.**

**
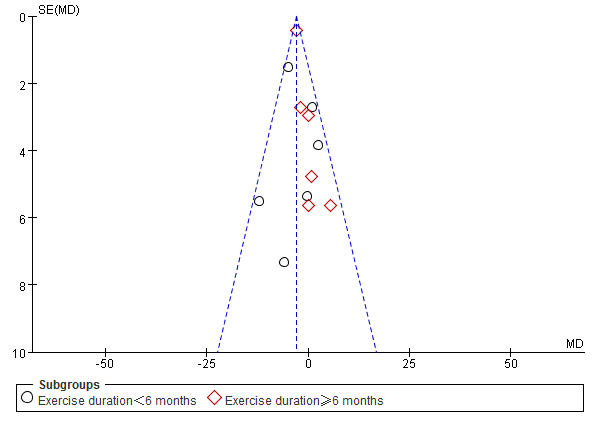
**

Supplement: Supplementary file 5 — Additional file 5: Fig. S4. Funnel plot of between-groups analysis for DBP. [file 12882_2019_1586_MOESM5_ESM.docx]

**Additional file 6:**

**Figure S5: Funnel plot of between-groups analysis for** **TC.**

**
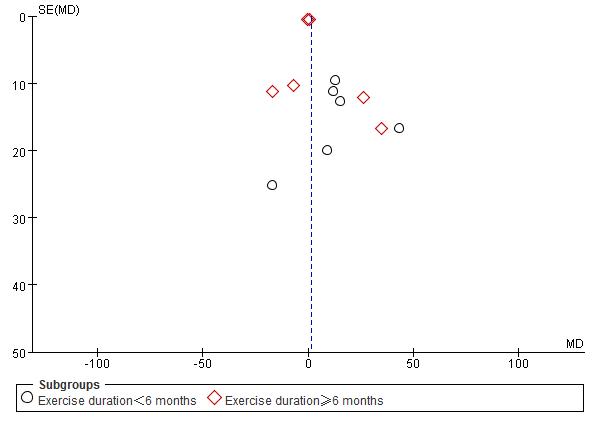
**

Supplement: Supplementary file 6 — Additional file 6: Fig. S5. Funnel plot of between-groups analysis for TC. [file 12882_2019_1586_MOESM6_ESM.docx]

**Additional file 7:**

**Figure S6: Funnel plot of between-groups analysis for TG.**


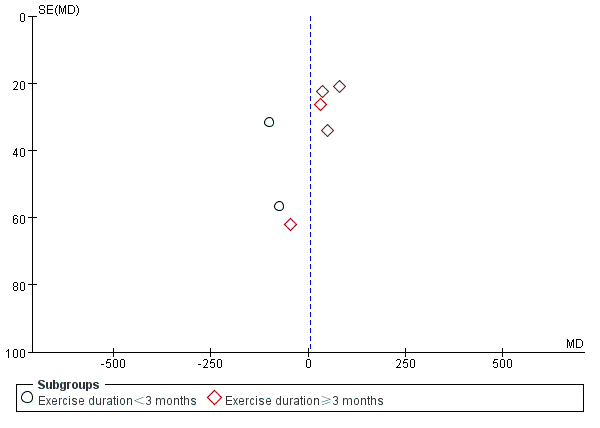

Supplement: Supplementary file 7 — Additional file 7: Fig. S6. Funnel plot of between-groups analysis for TG. [file 12882_2019_1586_MOESM7_ESM.docx]

**Additional file 8:**

**Figure S7: Funnel plot of between-groups analysis for HDL.**

**
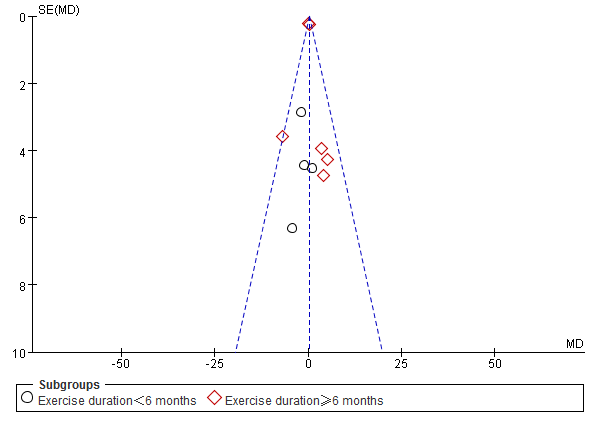
**

Supplement: Supplementary file 8 — Additional file 8: Fig. S7. Funnel plot of between-groups analysis for HDL. [file 12882_2019_1586_MOESM8_ESM.docx]

**Additional file 9:**

**Figure S8: Funnel plot of between-groups analysis for LDL.**

**
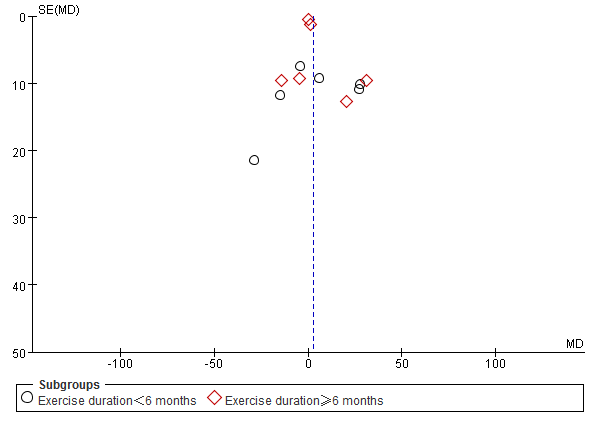
**

Supplement: Supplementary file 9 — Additional file 9: Fig. S8. Funnel plot of between-groups analysis for LDL. [file 12882_2019_1586_MOESM9_ESM.docx]

**Additional file 10:**

**Figure S9: Funnel plot of between-groups analysis for BMI.**

**
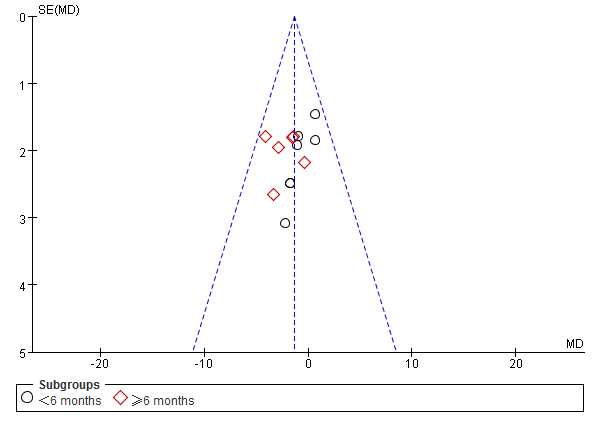
**

Supplement: Supplementary file 10 — Additional file 10: Fig. S9. Funnel plot of between-groups analysis for BMI. [file 12882_2019_1586_MOESM10_ESM.docx]
